# Complicated COVID-19 in pregnancy, maternal and neonatal outcomes: a case report

**DOI:** 10.11604/pamj.2022.41.191.31102

**Published:** 2022-03-09

**Authors:** Mouna Gara, Eya Sahraoui, Wafa Dhouib, Dhekra Toumi, Olfa Zoukar, Meriem Mehdi, Ali Jlali, Raja Faleh, Lotfi Grati

**Affiliations:** 1 Department of Anesthesia and Intensive Care “B”, Center of Maternity and Neonatology, Fattouma Bourguiba University Teaching Hospital, Monastir, Tunisia,; 2 Laboratory of Histology Embryology and Cytogenetics (LR 18 ES 40), Faculty of Medicine, University of Monastir, Avicenne Street, 5019 Monastir, Tunisia,; 3 Department of Preventive Medicine, University Hospital of Monastir, Monastir, Tunisia,; 4 Department of Gynaecology-Obstetrics, Center of Maternity and Neonatology, Fattouma Bourguiba University Teaching Hospital, Monastir, Tunisia

**Keywords:** COVID-19, pregnancy, respiratory distress syndrome, coagulopathy, case report

## Abstract

The novel coronavirus disease 2019 (COVID-19) has exposed vulnerable populations, including pregnant women, to an unprecedented public health crisis. According to recent data, pregnancy in COVID-19 patients is associated with increased hospitalization, admission to the intensive care unit (ICU) and intubation. It has been suggested that pregnancy induced immune responses and cardiorespiratory changes can exaggerate the course of the COVID-19. The present is a case of a pregnant woman who presented with critical respiratory failure secondary to COVID-19 resulted in her admission to the ICU and mechanical ventilator support. After childbirth, maternal outcomes were marked by disseminated intravascular coagulopathy and cardiopulmonary arrest on day thirty-four of admission. As to the neonatal outcome, a preterm female baby was transferred to the neonatal intensive care unit (NICU) and intubated immediately due to progressive respiratory distress. She was diagnosed with bacterial pneumonia with no evidence of COVID-19 and recovered after twenty-one days after NICU stay. This case showed that the maternal COVID-19 may lead to acute respiratory distress syndrome, coagulation dysfunction and preterm delivery. The risk of vertical transmission by SARS-CoV-2 is probably very low.

## Introduction

The novel coronavirus disease 2019 (COVID-19), caused by a new zoonotic coronary virus called SARS-CoV-2 (severe acute respiratory syndrome coronavirus 2), has spread rapidly from Wuhan to different areas of China and other parts of the world leading to a great threat to public health [1]. During the current pandemic, there is another special group that needs to be taken more care of, that is, pregnant women. During pregnancy, immunologic alterations and physiologic changes that affect respiratory and cardiovascular systems put women at increased risk for certain infections and associated complications [2]. Recent data from multiple studies showed the possible negative maternal and neonatal outcomes of COVID-19 infection during pregnancy such as maternal pneumonia and maternal death, sepsis, disseminated intravascular coagulopathy (DIC), preterm birth, neonatal pneumonia and neonatal death [3]. Thus, prevention and control of SARS-CoV-2 infection among pregnant women have become a major concern. In this study, we reported a case of a Tunisian pregnant woman in the third trimester, who had a rapid clinical deterioration from COVID-19-related acute respiratory distress syndrome (ARDS), showing coagulation impairment in the immediate post-partum period.

## Patient and observation

A 33-year-old gravida 2; para 1 woman with no medical history of cardiovascular nor other chronic diseases, at 28 weeks´ gestation, presented with a diagnosis of respiratory distress secondary to the novel COVID-19. The patient had a close contact with a confirmed COVID-19 case, developed symptoms (headache, myalgia, runny nose and joint pain) for one week before admission and reported being febrile with new onset of dyspnoea on the day before admission. The SARS-CoV-2 nasopharyngeal swab reverse transcriptase-polymerase chain reaction (RT-PCR) test was done and came back positive. Pregnancy was well monitored: biological and infectious checkups as well as ultrasounds were done, returning without abnormalities.

**Clinical findings:** on admission, the body mass index (BMI) was 36 kg/m^2^. Apart from obesity, her pregnancy had been without complications. The physical examination revealed a body temperature of 36.9°C, blood pressure of 120/80 mmHg, tachycardia with a heart rate of 112 beats per minute, respiratory rate of 40 breaths per minute with accessory muscles involvement and oxygen saturation of 92% with 12 litres per minute of oxygen on non-rebreather mask. Lung consultation revealed rhonchi over both lungs. Other laboratory tests showed elevation in inflammatory markers (C-reactive protein 144 mg/L). The initial obstetrics and gynaecological evaluation was unremarkable. The infant was eutrophic for the term (28 weeks´ gestation) without signs of suffering or detectable abnormality. The patient received treatment with intravenous antibiotics (Azithromycin, Amoxicillin and Clavulanic Acid…), corticosteroid therapy (Dexamethasone), vitamin C and zinc, and anticoagulant therapy (Enoxaparin).

**Figure 1:**
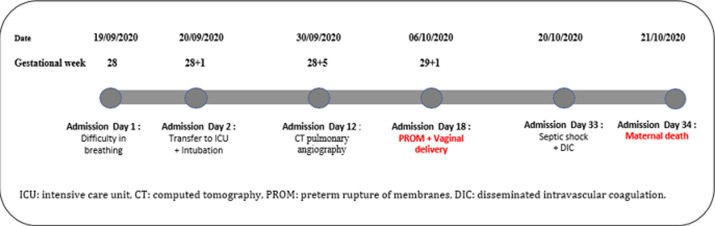
timeline of the patient’s admission

**Timeline:** her condition deteriorated drastically on day two of admission because of worsening respiratory distress and increased oxygen requirements. The patient was subsequently transferred to the ICU, sedated and intubated, and invasive mechanical ventilation was started with protective settings (Figure 1).

**Figure 2:**
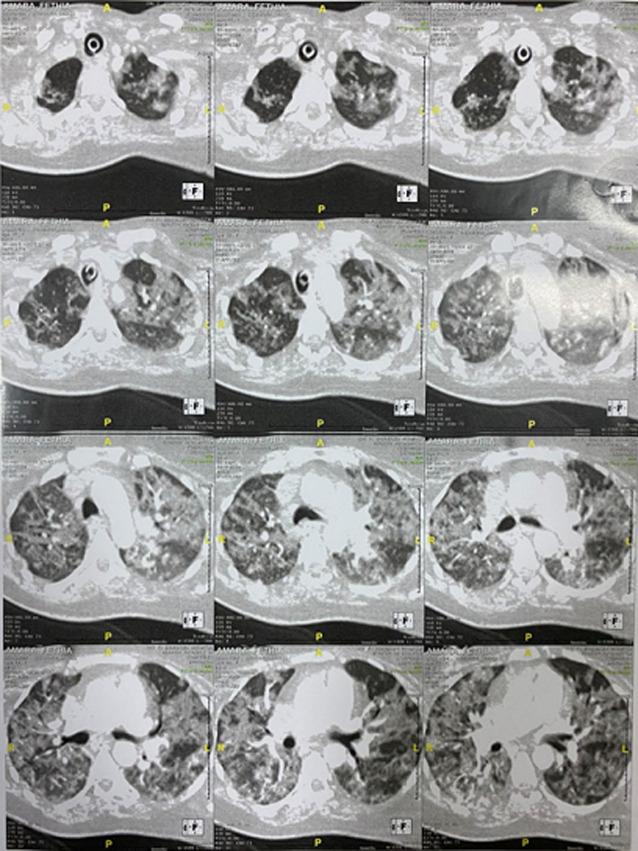
computed tomography pulmonary angiography of the pregnant woman, bilateral diffuse ground-glass opacities, that have a mid and upper zone distribution, reaching more than 75% of the pulmonary parenchyma and associated with subpleural consolidations which predominated in the lung bases and no signs of pulmonary embolism (twelve segments from the same session)

**Diagnostic assessment:** on day twelve of admission, a computed tomography (CT) pulmonary angiography was practised in order to evaluate the aggravation of pulmonary lesions. It showed bilateral diffuse ground-glass opacities, that have a mid and upper zone distribution, reaching more than 75% of the pulmonary parenchyma and associated with subpleural consolidations which predominated in the lung bases, but no signs of pulmonary embolism (Figure 2). Patient was O Rhesus D positive. Laboratory tests are shown in (Table 1).

**Table 1:** maternal laboratory results during admission

Variable	Reference range	Admission day1 (19/09/2020)	Admission day 2 (20/09/2020) prior to intubation
Hemoglobin (Hb) g/L	117-153	118	-
Hematocrit %	37-46	33	-
Platet count × 10^9^/ L	165-387	157	-
White cell count × 10^9^/ L	3.5-8.8	7.24	-
Neutrophil count × 10^9^/ L	1.8-7.5	2.8	-
Lymphocyte count × 10^9^/ L	1.0-4.0	1.9	-
CRP mg/L	< 5	144	-
Glucose mmol/L	4.2-6.0	4.3	
ASATU I/L	13-35	13	-
ALATU I/L	7-56	6.23	-
Bilirubin μmol/L	5-25	3	-
LDHμk at/L	1.8-3.4	2.3	-
Creatinine μmol/L	45-90	50	-
Urea mmol/L	2.6-6.4	1.3	-
Uric acid μmol/L	155-350	382	-
Sodium mmol/L	137-145	128	-
Potassium mmol/L	3.5-4.4	3.1	-
Arterial blood gases			
pH	7.35-7.45	7.44	7.34
pCO_2_ mmHg	35-45	28	35
pO_2_ mmHg	80-100	79	51
HCO_3_ mmol/L	21-27	18	18.9
SaO_2_ %	97-100	95	88

CRP: C-reactive protein, ASAT: Aspartate aminotransferase, ALAT: Alanine aminotransferase, LDH: Lactate Dehydrogenase; pCO_2_: Partial pressure of carbon dioxide, pO_2_: Partial pressure of oxygen, HCO_3_-: bicarbonate, SaO_2_: arterial saturation of oxygen.

**Therapeutic Intervention:** despite ventilator support, the computed arterial oxygen pressure on oxygen inspired fraction ratio (PaO_2_/FiO_2_) was 75 mmHg, indicative of severe ARDS. The severe hypoxemia was deemed potentially harmful to the fetus, and various options were considered to improve the patient´s oxygenation. A neuromuscular block was administered. The agent used was Cisatracuruim at a dose of 37.7 mg/h. She also received inhaled nitric oxide at a dose of 0.4 to 0.6 part per million (ppm) but considering the lack of improvement in oxygen saturation despite mechanical ventilation with 100% fraction of inspired oxygen (FiO_2_) and deep sedation, she was placed in prone position successfully performed with the use of supports and pads beneath shoulders and hips, to prevent aortocaval compression.

**Follow-up and outcomes:** maternal respiratory status gradually improved after optimizing ventilation and starting a 12-hour daily cycle of pronation. However, the patient remained unresponsive after discontinuation of sedation and paralysis and repeatedly failed attempts at weaning from mechanical ventilation. Meanwhile, fetal well-being was reassessed reassuring. Daily fetal monitoring showed no signs of fetal distress, with a stable fetal cardiac rhythm around 140 beats per minute. On day eighteen of admission, the patient experienced acute tachycardia (110 beats per minute) despite ongoing deep sedation. On exam, her membranes had spontaneously ruptured, with baby's head reaching the vaginal opening. Anaesthesiology, obstetric, neonatal, and medical intensive care teams were alerted and all personnel involved wore protective gear including gown, N95 mask, eye protection and gloves. Induction of labour with IV oxytocin (10 UI) was started, with initial plan to attempt vaginal delivery with the assisted second stage. An uncomplicated delivery was completed within 15 minutes. A preterm female baby was delivered with birth weight, 1200g. Apgar's scores were 7, 8 and 8 at 1, 5 and 10 minutes, respectively. She was transferred to the neonatal intensive care unit (NICU) and intubated immediately due to progressive respiratory distress. Neonate was tested for SARS-CoV-2 which turned out negative. However, she was diagnosed with bacterial pneumonia and then provided with adequate anti-biotherapy.

On the other hand, the patient´s condition deteriorated shortly after the delivery. She sustained a post-partum haemorrhage of more than 500 mL controlled with uterotonic agents, 2g tranexamic acid, and blood transfusion. The haemoglobin level reached the lowest value (<7g/dL) with elevated prothrombin time and thrombocytopenia, indicative of disseminated intravascular coagulation (DIC). The patient remained intubated, with increasing oxygen requirements in the post-partum period and the blood pressure was maintained within the normal range by noradrenalin continuous infusion of 3.5 mg/h. General condition has worsened. Her ARDS acutely persisted (PaO_2_/FiO_2_ <100 and arterial oxygen saturation (SaO_2_) of 60 %). Her DIC persisted as well. On day thirty-four of admission, the patient progressed to profound bradycardia, had cardiopulmonary arrest and died after failed resuscitative efforts. As to the neonatal outcome, the baby was initially improved to be extubated. Twenty-one days after NICU stay, she was discharged after showing sufficient recovery in both clinical and para clinical parameters.

## Discussion

We reported a critical case of COVID-19 in pregnancy, which led to acute respiratory distress syndrome, disseminated vascular coagulopathy (DIC) and preterm delivery with no vertical transmission. This case adds to the growing body of evidence that raises concerns about the potential negative outcomes of COVID-19 during pregnancy and more particularly in thromboembolic complications, especially since there was no rich bibliography concerning this fatal outcome. While most mothers with COVID-19 infection have mild symptoms which resolve without treatment, a number of cases have recently emerged in the literature, where mothers have required ICU admission due to hypoxemic respiratory failure, with extracorporeal membrane oxygenation [4]. Immunologic alterations and physiologic changes that affect respiratory, cardiovascular, and other organ systems during pregnancy can exaggerate the course of the COVID-19 pneumonia [2].

In the current reported case, the patient presented with a clinical picture of severe acute respiratory illness. The patient´s current pregnancy was considered high-risk due to her high BMI which may have largely contributed to the development of ARDS in the setting of COVID-19 pneumonia. Severe hypoxia requiring intensive care support may be explained by the spread of the coronavirus 2 through the bloodstream and mainly in the lungs, which occurs around 7-14 after the onset of the symptoms. At this time, the virus starts a second attack, which is also the main cause of the aggravation of symptoms [5]. Pulmonary lesions became worse, and chest CT scans imaging changes consistent with COVID-19. At this stage, the peripheral blood lymphocytes decrease significantly, involving both T and B-lymphocytes. Inflammatory factors in peripheral blood are increased [5].

Respiratory management of severe COVID-19-related ARDS followed the guidelines of the ARDS caused by viral pneumonia with protective ventilation including low tidal volume, high positive end expiratory pressure (PEEP), deep sedation, prone positioning, neuromuscular blocking agents and inhaled nitric oxide. During ICU stay, the patient´s condition has worsened due to a post-partum haemorrhage after a vaginal delivery, rapidly followed by thromboembolic complications. In the literature, one case report had described mortality in a woman at 29 week gestation with COVID-19 due to thromboembolic complications [6]. It is believed that COVID-19 can activate the coagulation cascade through various mechanisms, leading to severe hypercoagulability [5]. Given that pregnancy itself is a hypercoagulable state and coagulopathy is linked to a poorer diagnosis, it has been suggested that COVID-19 infection during pregnancy is associated with high risk of maternal thrombotic complications [6]. Outside of pregnancy, anticoagulation of coagulopathic, septic patients is recommended. Early anticoagulation may block clotting formation and reduce microthrombus, thereby reducing the risk of major organ damages [5].

Laboratory features noted elements of DIC (low haemoglobin level, elevated prothrombin time and a decreasing platelets count). DIC occurs when the viral damage is associated with endothelial damage that may also induce ischaemia of tissues and organs [7]. In fact, the virus penetrates human target cells mainly by binding angiotensin-converting enzyme 2, which is highly expressed in alveolar and cardiac cells, but also in the vascular endothelium [8]. We highlight a possible link between delivery and rapid maternal deterioration, with coagulation disorders. We suggest that delivery resulted in adverse maternal outcome and mortality. On the other hand, it was found that delivery may improve respiratory distress and time to recovery in COVID-19-related ARDS during pregnancy [9].

Considering the neonate, COVID-19 may constitute a threat of premature delivery. Among pregnant women with SARS-CoV-2, preterm birth is reported 47 % (15/32) [10, 11]. Furthermore, we found no evidence for any vertical transmission of COVID-19 between mother and the baby, which is consistent with a systematic review documenting no evidence of virus transmission in women with COVID-19 [12]. As yet, there is no clear-cut evidence about this subject. The risk of ingestion or aspiration of cervicovaginal secretions or contact with perineal infected tissue is higher with vaginal delivery. However, virus transmission didn´t occur in the case that we reported. We can only speculate whether this indicated that vertical transmission of SARS-CoV-2 is low for vaginal delivery.

## Conclusion

The upper is a description of a severe case of maternal COVID-19 during the third trimester of pregnancy which led to severe ARDS, disseminated vascular coagulopathy (DIC) and preterm delivery with no vertical transmission. Considering the importance of this ongoing global public health emergency, case reports and observational studies are still necessary to understand and determine the risk of thromboembolic complications in pregnant women.
